# Abdominoplasty in the Presence of a Stoma for Functional and Aesthetic Indications

**DOI:** 10.1093/asjof/ojad009

**Published:** 2023-02-02

**Authors:** Joseph M Firriolo, Ashley K Truong, Heath J Charvet

**Affiliations:** Division of Plastic and Reconstructive Surgery, University of California Davis, Sacramento, CA, USA; Division of Plastic and Reconstructive Surgery, University of California Davis, Sacramento, CA, USA; private practice in New Orleans, LA, USA

## Abstract

**Background:**

Abdominoplasty is widely available; however, patients with abdominal stomas appear to be relatively undertreated. Apprehension to offer abdominoplasty in the presence of a stoma may be secondary to the fear of surgical site infection and stoma compromise.

**Objectives:**

To demonstrate the feasibility and safety of abdominoplasty in the presence of an abdominal stoma for both functional and aesthetic indications and to define perioperative protocols to reduce the risk of surgical site infection in this patient population.

**Methods:**

The authors present 2 patients with stomas who underwent abdominoplasty. Patient 1 was a 62-year-old female with a history of urostomy formation and weight loss. She had a fold of skin overhanging her ostomy site, making it difficult to maintain a seal on her urostomy bag. She underwent fleur-de-lis abdominoplasty and urostomy revision. Patient 2 was a 43-year-old female with a history of end ileostomy formation, who requested cosmetic abdominoplasty to address postpartum abdominal changes; she had no functional stoma-related complaints. Abdominoplasty, flank liposuction, and ileostomy revision were performed.

**Results:**

Both patients were satisfied with their aesthetic and functional outcomes. There were no complications and no instances of stoma compromise. At follow-up, Patient 1 reported a complete amelioration of her urosotomy appliance issues.

**Conclusions:**

Abdominoplasty may confer both functional and aesthetic benefits to patients with abdominal stomas. The authors present peri- and intraoperative protocols, both to prevent stoma compromise and to reduce the risk of surgical site infection. The presence of a stoma does not appear to be an absolute contraindication to cosmetic abdominoplasty.

**Level of Evidence: 5:**

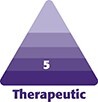

Abdominoplasty is one of the most common aesthetic procedures; a total of 242,939 abdominoplasties were performed by ASAPS members in 2021 (a 49% increase from the previous year).^1^ Abdominoplasty is powerful in its ability to improve abdominal contour by addressing excess skin, excess fat, and rectus muscle diastasis, thereby conferring improvements in patient self-esteem, body image, and quality of life. Despite the widespread availability of this operation, patients with abdominal stomas appear to be relatively undertreated. Apprehension to offer abdominoplasty in the presence of a stoma may be secondary to the fear of stoma compromise as well as concern for an increased risk of surgical site infection. Previous authors have reported success in the so-called functional abdominoplasty among patients with stomas.^2-6^ Specifically, abdominoplasty has proven valuable in addressing abdominal contour irregularities that may cause difficulty maintaining appliances, stool/urine leakage, and skin irritation. In the present study, the authors report the case of 2 patients with abdominoplasty in the presence of a stoma: 1 “functional” abdominoplasty (with urostomy revision) and 1 purely cosmetic abdominoplasty (with ileostomy revision). To our knowledge, the present study is the first in which authors document pure cosmetic abdominoplasty in a patient with a stoma. This report is also the first of its kind in which authors define perioperative protocols to reduce the risk of surgical site infection in this patient population.

## CASE PRESENTATIONS

This study received Institutional Review Board exemption by the University of California, Davis Institutional Review Board. Written consent was provided, by which the patients agreed to the use and analysis of their data. All procedures (including stoma modification and abdominoplasty) were performed by the senior author of the present study (H.J.C.).

### Patient 1: Functional Abdominoplasty in the Presence of Urostomy

**Figure 1.**
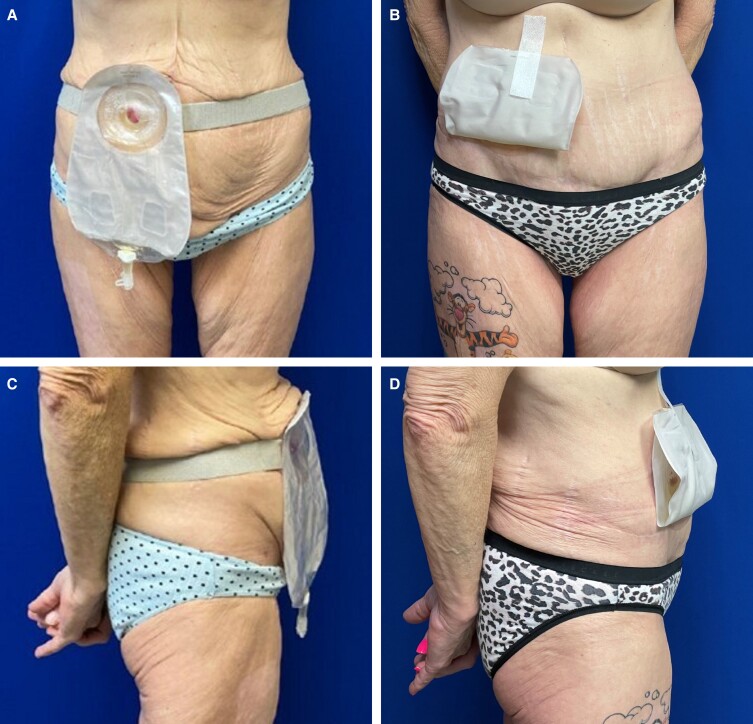
Patient 1: a 62-year-old female shown at (A) frontal view preoperatively; (B) frontal view, 2 years postoperatively, following fleur-de-lis abdominoplasty and urostomy revision; (C) lateral view preoperatively (note the superior fold of skin overriding the urostomy appliance); and (D) lateral view, 2 years postoperatively, following fleur-de-lis abdominoplasty and urostomy revision.

Patient 1 is a 62-year-old female with a medical history significant for scleroderma (on hydroxychloroquine and mycophenolate) and recurrent bladder cancer, necessitating neobladder formation with an ileal conduit and urostomy. Over the course of the antecedent 12 years, through diet and exercise, her weight decreased from 113.6 kg to 55.5 kg (BMI 39.2-19.1 kg/m^2^). She did not have any other significant comorbid conditions; patient history was negative for diabetes mellitus, smoking, and hypertension. She presented to our clinic with a chief complaint of urostomy appliance dysfunction secondary to abdominal skin laxity. Specifically, she reported a superior fold of skin overhanging her ostomy site, making it difficult to obtain and maintain a seal on her urostomy bag (Figure 1). She reported urine leakage, frequent appliance changes, skin irritation, and social embarrassment. On examination, she had abdominal skin excess in both the vertical and the horizontal dimensions as well as rectus diastasis.

The patient was consented for abdominoplasty with urostomy takedown and replacement. Mycophenolate was discontinued 7 days preoperatively. Communication with the prescribing rheumatologist is paramount and timing of discontinuation of immunosuppressive drugs should be approached and determined on a patient-by-patient basis. In the operating room, the patient was prepped and draped in the supine position. First, the urostomy was closed with a running locking 2-0 silk sutures. A cuff of skin was then excised around the urostomy and dissection was taken to the fascia and the urostomy was freed. A sterile glove was secured over the opening to collect any urine spillage. The lower abdominoplasty incision was made and the abdominoplasty flap was elevated superiorly in a standard fashion (to the level of the costal cartilage and xiphoid), with maintenance of the umbilicus. The superior incision was then made and a 415 g specimen removed. Although this patient had mild rectus diastasis, fascial plication was not performed as there was minimal fascial laxity and there were intraoperative concerns of kinking her ostomy at the fascial opening. Antibiotic irrigation was performed, hemostasis achieved, and two 19 Fr Blake drains were placed. The incision was approximated with towel clips, but the patient's midline abdominal scar prevented direct skin apposition. A decision was made to excise this scar in a fleur-de-lis style (also serving to eliminate horizontal abdominal skin excess). The vertical incision was closed and room was made to incorporate the umbilicus. The area for the urostomy was marked on the overlying skin and an oval of skin was excised; dissection was carried through the fat to the fascia and the urostomy delivered. The sterile glove was removed from around the urostomy and inset was performed with 3-0 Vicryl brooking sutures (Ethicon, Raritan, NJ). A urostomy bag was applied and urine could be seen collecting. The horizontal incision line was closed, steri-strips applied, and drains placed on the bulb suction. The patient was placed in an abdominal binder for 6 weeks. She was then placed on a 5-day postoperative course of ciprofloxacin and the drains were removed when outputs were less than 20 mL/day for 2 consecutive days. She followed the expected postoperative course without complication. Upon follow-up, she reported a complete amelioration of her urostomy appliance issues, including a resolution of leakage and far less frequent appliance changes (Figure 1). She also expressed satisfaction with her improved body image and quality of life.

### Patient 2: Cosmetic Abdominoplasty in the Presence of Ileostomy

**Figure 2.**
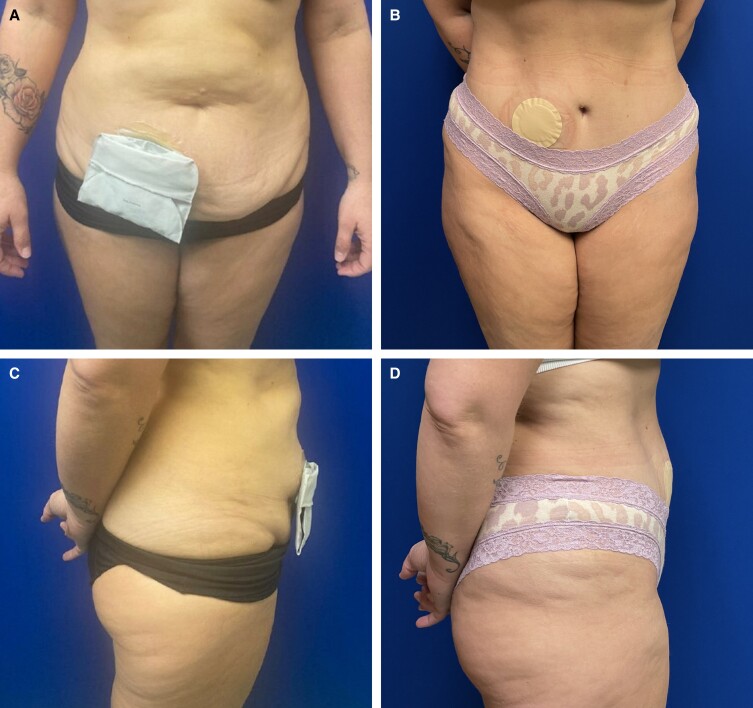
Patient 2: a 43-year-old female shown at (A) frontal view preoperatively; (B) frontal view, 1½ years postoperatively, following abdominoplasty, flank liposuction, and ileostomy revision; (C) lateral view preoperatively; and (D) lateral view, 1.5 years postoperatively, following abdominoplasty, flank liposuction, and ileostomy revision.

Patient 2 was a 43-year-old female with inflammatory bowel disease who underwent total colectomy with end ileostomy 10 years prior to consultation. She presented with excess abdominal skin and rectus diastasis because of a combination of pregnancy and weight loss (BMI 26.5 kg/m^2^) (Figure 2). She did not have any other significant comorbid conditions; patient history was negative for diabetes mellitus, smoking, and hypertension. Her desire to pursue abdominoplasty was purely cosmetic in nature; there were no patient-reported stoma complaints. Of note, the patient recounted that she had seen several surgeons who all declined to attempt cosmetic abdominoplasty because of the presence of an ileostomy. The patient was consented for abdominoplasty, suction-assisted lipectomy (flanks, upper abdomen, and mons), and ileostomy revision. This case was considered purely aesthetic in nature; there was no submission for insurance authorization for any component of this procedure.

Preoperatively—in anticipation of ostomy dissection, movement, and maturation—we had the patient undergo bowel preparation. The patient was given GoLytely (Braintree Laboratories Inc., Braintree, MA) the day prior to the procedure and instructed to consume 2 L between 12 pm and 2 pm. Oral antibiotics were commenced after completion of the mechanical bowel preparation. If the patient had a colostomy and not an ileostomy, the complete 4 L dose of GoLytely would be used with a goal of clear output. The antibiotic regimen consisted of 3 doses of 1000 mg of Neomycin and 3 doses of 500 mg metronidazole (at 2 pm, 3 pm, and 10 pm) the day prior to surgery. The patient was instructed to stay hydrated and consume a protein and carbohydrate-rich diet for 2 to 3 days prior to the operation to promote an anabolic state prior to surgery. Additionally, the patient was instructed to drink two 20 oz electrolyte drinks (eg, Gatorade) between the completion of bowel prep and midnight.

In the operating room, the patient was positioned supine, prepped, and draped. Tumescent solution was infiltrated into the liposuction sites. Before making any incisions, the end of the ostomy was oversewn with a running locking 0 silk suture to prevent any gross spillage. The ostomy was located in the area of the abdomen to be resected in abdominoplasty. A 5 mm skin cuff was excised around the ostomy and dissection down the level of the fascia was performed sharply with Metzenbaum scissors to reduce thermal injury to the intestine; a sterile glove was secured over the opening. The umbilicus was isolated on its stalk taking care to preserve some fat for vascularity. The flap was then incised inferiorly and elevated superiorly taking care to isolate and protect the ileostomy. The flap dissection proceeded superiorly to the costal margin laterally and to the xiphoid medially.

Fascial plication was done superiorly and inferiorly in 2 layers. The ostomy penetrated the fascia near the umbilical stalk approximately 1 cm lateral to the right and 1 cm inferior. The fascial entrance of the ostomy determines the appropriateness of plication. Colostomy location typically occurs in the left lower quadrant of the abdomen, and ileostomy placement in the right lower quadrant, both allowing traditional midline plication. In this case, the proximity of the ostomy to the umbilicus prevented safe midline plication to the level of the umbilicus. Plication was instead performed superiorly and inferiorly in the midline. To further improve contour and fascial tightening, 3 interrupted 0 Ti-Cron figure-of-8 sutures (Medline Industries, Inc., Northfield, IL) were placed bilaterally over the linea semilunaris. The abdomen was irrigated with antibiotic solution, hemostasis achieved, and two 19 Fr Blake drains were placed.

Once semifowler position was achieved, the amount of abdominal skin that could be safely removed was marked and the patient was stapled together. The umbilical and ostomy skin openings were marked. The abdomen was then closed in 3 layers, 2 drains were placed, and the umbilicus inset was performed. A total of 1000 drapes were used to isolate the incisions away from the ostomy (see photograph). Ostomy location was designed such that the new stoma would be placed cephalad enough to allow appliance application without inclusion of the transverse scar, but low enough to meet patient satisfaction. In this case, preoperative discussion included the desire to keep the ostomy covered with bikini bottoms. Anatomic location in relation to her anterior superior iliac spine was used to help identify the area for stoma maturation. The ostomy was delivered through the new skin opening and a 2 mm cuff of bowel was removed to include the oversewn mucosa. The inset was completed in a standard brooking fashion with a healthy viable stoma. At the termination of the case, a small finger was used to probe the ostomy and confirm no kinks existed within the ostomy through the level of the fascia.

The patient was placed in an abdominal binder for 6 weeks. She was placed on a 5-day postoperative course of ciprofloxacin and metronidazole. Drains were removed when outputs were less than 20 mL/day for 2 consecutive days. She had no complications, no stoma compromise or dysfunction, and stated that her cosmetic outcome far exceeded her expectations (Figure 2).

For the above case presentations, both patients were placed on the enhanced recovery after surgery (ERAS) protocol. Preoperative counseling is an important component of this protocol and involves nutrition education/optimization, setting pain expectations, and a thorough discussion of postoperative instructions. Chlorhexidine body wash was provided, and the patients were instructed to use the body wash 2 nights preoperatively, 1 night preoperatively, and finally on the morning of the operation. Postoperatively, the patients were discharged with sequential compression devices that were worn continuously for 7 days; ambulation was encouraged. Caprini scores are calculated for all patients and chemoprophylaxis is added when scores are 6 or greater. Here, multimodal analgesia was established with scheduled acetaminophen (oral, 1000 mg every 6 h), scheduled gabapentin (oral, 300 mg 3 times daily for 3 days preoperatively and 3 days postoperatively), and opioid medications as required for breakthrough pain. Nonsteroidal anti-inflammatory drugs (NSAIDs) normally feature in our group's ERAS protocol, but here, they were not prescribed because of each patient's relative contraindication, that is, a history of NSAID-induced gastrointestinal upset in the setting of scleroderma (Patient 1) and a history of inflammatory bowel disease and ileostomy (Patient 2).

## DISCUSSION

We report successful abdominoplasty—without complication—in 2 patients with stomas: 1 “functional” abdominoplasty (with urostomy revision) and 1 cosmetic abdominoplasty (with ileostomy revision). We also define perioperative protocols to reduce the risk of surgical site infection in this patient population.

Ostomy dysfunction is a significant detriment to the quality of life. Patients may report issues in maintaining ostomy appliances, the need for frequent appliance changes, leakage of stool/urine, skin irritation, and social embarrassment. Avoidance of these issues is underpinned by meticulous preoperative planning of stoma location.^4^ Enterostomal therapists will mark patients for stoma placement by factoring in body habitus, skin folds, scarring, bony prominences, rectus abdominis muscle anatomy, and a patient's belt line. Most cases of patients with ostomy dysfunction are amenable to local treatment options, namely patient-specific tailoring of ostomy devices, formal stoma revision/relocation, and peristomal fat excision and liposuction.^3,7^ Despite these various options, the concept of “functional” abdominoplasty has emerged in the literature because of its unique advantages in the setting of ostomy dysfunction.^2-6^

In contrast to the above-listed local peristomal measures, functional abdominoplasty can address abdominal contour irregularities, in the setting of both weight gain and weight loss. Weight loss may result in skin redundancy and folds that cause difficulty with device application as well as ostomy retraction and/or kinking. Likewise, weight gain may also result in ostomy retraction secondary to an increase in abdominal girth. Substantial weight change is not uncommon among patients with stomas, especially among those with prolonged illness, steroid usage, and bowel resection(s).^2-4^ Patients with multiple abdominal operations also carry a significant scar burden. Scarring may directly cause ostomy retraction; it can also make it difficult to resite a stoma. For such patients, abdominoplasty may recruit nonscarred, upper abdominal tissue for stoma resiting.^5^

Patient 1 in our series underwent abdominoplasty in the setting of urostomy and massive weight loss. Her primary complaint was redundant overhanging skin causing urine leakage and issues with ostomy appliance maintenance. Similar to the case series by Mickute and colleagues, our patient experienced a resolution of all ostomy concerns and was satisfied with her improved cosmesis. Although not applicable to Patient 1, it has also been highlighted that peristomal hernias may also be a cause of stoma dysfunction and may be easily repaired through the wide access offered by abdominoplasty flap elevation.^4,8^

“Functional” abdominoplasty is modestly represented in the literature, with publications limited to case reports and case series.^2-6^ Among these reports, authors have demonstrated the safety of abdominoplasty and justify the decision to operate based on medical necessity, ie, to address ostomy dysfunction when local measures will not suffice. Although concerns of surgical site infection and ostomy injury may deter some surgeons from offering abdominoplasty in this population, for certain surgeons and patients, the gains in ostomy function apparently outweigh the perceived risks of abdominoplasty in this group. It is then interesting to note that the present study is the first among English-language publications in which authors report abdominoplasty in a patient with a stoma for a purely cosmetic purpose (ie, Patient 2).

Patient 2 recounted that she was declined abdominoplasty by several surgeons before her initial consult with our practice. She reported body image deficits related to both her ileostomy and postpartum abdominal changes. Body image concerns in the setting of stomas are well documented; it is plausible that aesthetic surgery of the trunk may improve self-esteem for well-selected patients with stomas.^9,10^ Furthermore, even if primarily for functional reasons, abdominoplasty enhances cosmesis in ways that cannot be achieved with local stoma revision options. Distinguishing features of abdominoplasty include improved abdominal contour, excision of scarred skin, and the ability to correct rectus diastasis.

Our success and comfort with abdominoplasty among patients with stomas may be partially attributable to our perioperative protocols. For instance, we are not aware of any previous reports that detail preoperative bowel prep and perioperative targeted antibiotic regimens for patients with stomas undergoing abdominoplasty. Implementation of these protocols may reduce the risk of surgical site infection.

Limitations of this study must be acknowledged, the most salient being that this study is a case series of 2 patients. In addition, the techniques we discuss may not be generalizable to all plastic surgical practices. Importantly, we recommend that ostomy revision be performed by a surgeon with the appropriate training/experience. The senior author of this study (H.J.C.) is board-certified in general surgery. Given the trend toward integrated training models in the United States, many plastic surgeons may benefit by performing these procedures in collaboration with general surgeons or urologists, as indicated. To that end, it may be difficult to offer these procedures in private practice unless a surgeon is personally comfortable with their knowledge and skill regarding ostomy revision.

## CONCLUSIONS

Abdominoplasty may confer both functional and aesthetic benefits to patients with abdominal stomas. We present our peri- and intraoperative approach to abdominoplasty in the presence of a stoma, both to prevent stoma compromise and to reduce the risk of surgical site infection. The presence of a stoma does not appear to be an absolute contraindication to cosmetic abdominoplasty. Further research is indicated to confirm the safety of abdominoplasty among this population as well as to further refine perioperative protocols.
